# Dual-mobility cup total hip arthroplasty improves the quality of life compared to internal fixation in femoral neck fractures patients with severe neuromuscular disease in the lower extremity after stroke: a retrospective study

**DOI:** 10.3389/fsurg.2023.1120273

**Published:** 2023-04-17

**Authors:** Chaolun Liang, Bojian Chen, Zhifeng Hu, Xing Li, Yongming Huang

**Affiliations:** ^1^ The 2nd Clinical Medical College, Guangzhou University of Traditional Chinese Medicine, Guangzhou, China; ^2^ Department of Orthopaedic Surgery, The Second Affiliated Hospital of Guangzhou University of Chinese Medicine, Guangzhou University of Chinese Medicine, Guangzhou, China

**Keywords:** dual-mobility cup total hip arthroplasty, internal fixation, quality of life, stroke, severe neuromuscular dysfunction

## Abstract

**Background:**

This study aimed to demonstrate that dual-mobility cup total hip arthroplasty (DMC-THA) can significantly improve the quality of life (QOL) of elderly femoral neck fracture patients with severe neuromuscular disease in unilateral lower extremities due to stroke hemiplegia compared to internal fixation (IF).

**Methods:**

Fifty-eight cases of severe neuromuscular disease in the unilateral lower extremities with muscle strength < grade 3/5 due to stroke were retrospectively examined From January 2015 to December 2020. Then, patients were divided into DMC and IF groups. The QOL was examined using the EQ-5D and SF-36 outcome measures. The physical and mental statuses were assessed using the Barthel Index (BI) and e Fall Efficacy Scale-International (FES-I), respectively.

**Results:**

Patients in the DMC group had higher BI scores than those in the IF group at different time point. Regarding mental status, the FES-I mean score was 42.1 ± 5.3 in the DMC group and 47.3 ± 5.6 in the IF group (*p* = 0.002). For the QOL, the mean SF-36 score was 46.1 ± 18.3 for the health component and 59.5 ± 15.0 for the mental component in the DMC group compared to 35.3 ± 16.2 (*p* = 0.035), and 46.6 ± 17.4 (*p* = 0.006) compared to the IF group. The mean EQ-5D-5L values were 0.733 ± 0.190 and 0.303 ± 0.227 in the DMC and IF groups (*p* = 0.035), respectively.

**Conclusion:**

DMC-THA significantly improved postoperative QOL compared to IF in elderly patients with femoral neck fractures and severe neuromuscular dysfunction in the lower extremity after stroke. The improved outcomes were related to the enhanced early, rudimentary motor function of patients.

## Introduction

Stroke is the second leading cause of disability and death worldwide, especially among the elderly, with projected mortality rates of 24.9% by 2030 (1). Up to 88% of stroke patients experience hemiplegia (2). Multiple studies have shown that people with hemiplegia after a stroke have lower bone mineral density and bone strength than the general elderly population (3, 4), thereby increasing the risk of femoral neck fracture (5).

Hip arthroplasty (HA) can substantially improve patients' function, quality of life (QOL), and survival, recognized as the primary therapeutic approach for femoral neck fractures. However, HA is restricted to individuals in good physical condition. Clinically, some elderly stroke patients have severe neuromuscular dysfunction with muscle strength < grade 3/5 (Lovett scale) in one lower extremity. The reduction of periprosthetic muscle and fascia tension increases the risk of prosthesis dislocation and loosening, resulting in poor QOL after surgery (6, 7). Consequently, most doctors believe that internal fixation (IF) is more appropriate than HA for these patients with severe neuromuscular illness in the unilateral lower extremity (muscle strength < grade 3/5) (8). However, for IF, patients should remain in bed long after surgery, which is not conducive to enhancing function and QOL (9, 10).

Currently, how to improve the QOL of people suffering from severe hemiplegia remains unclear. Dual-mobility cup total hip arthroplasty (DMC-THA) might be a viable option (11). Bousquet first presented the idea of dual-mobility hip articulations in 1974 (12). This unique artificial joint system adds a high molecular polyethylene liner between the acetabular cup and femoral head to create two mobility interfaces that can increase the hip joint's range of motion and decrease dislocation risk (13). DMC-THA has successfully treated femoral neck fractures in older patients with neuromuscular diseases. However, most studies were concentrated on patients more prone to recover quickly (14).

Therefore, this study aimed to show that DMC-THA, instead of IF, can significantly improve the QOL for senior femoral neck fracture patients with severe neuromuscular dysfunction in one lower extremity from hemiplegia caused by stroke.

## Materials and methods

Herein, we retrospectively analyzed the clinical data of femoral neck fracture patients with severe stroke hemiplegia admitted to the Guangdong Hospital of Traditional Chinese Medicine in Guangzhou, China, between January 2015 and December 2020, with a minimum 1-year follow-up. The inclusion criteria were: femoral neck fracture and stroke hemiplegia, unilateral lower extremity muscular strength < grade 3/5 (Lovett scale) (15), and treatment at our institution. The exclusion criteria included bilateral lower extremity impairment, revision arthroplasty, pathological fractures, less than one year of follow-up, and lack of specific information. We retrieved 233 femoral neck fracture cases with stroke hemiplegia *via* the electronic medical record (EMR) system. Then, 163 patients who did not fit the inclusion criteria were excluded, seven patients were lost to follow-up or had insufficient data, and five patients passed away. Finally, 58 cases were included for analysis. All cases were separated into two groups based on the surgical technique. This study was authorized by the Investigational Ethics Review Board (Guangdong Hospital of Traditional Chinese Medicine). An exemption from informed consent was acquired from the board.

The patient data consisted of two sections: hospitalization and follow-up information. Hospitalization information comprised demographic characteristics, such as age, gender, and BMI. Clinical data included the kind of femoral neck fracture, activities of daily living (ADL) at admission and discharge, operative blood loss, and surgery time. The ADL was evaluated using the Barthel index (BI). Patients unable or unwilling to attend the hospital were contacted by telephone (16–18). Follow-up records included follow-up time, postoperative complications, reoperation, rehabilitation time, initial time to get off the ground, ADL follow-up assessment, fear of falling (FOF), and QOL. The mortality rate was excluded since most participants were elderly with a high risk of natural death (19). The FOF was assessed with the Fall Efficacy Scale-International (FES-I) to determine the psychological state of patients (20, 21). The QOL was determined with the SF-36 Health Survey and EQ-5D-5L score (22–24). EQ-5D-5L utility scores derived from the Chinese Time-Trade Off (TTO) were used for additional analysis (25). All patient data, including baseline characteristics, clinical data, and follow-up information, were compiled by a research assistant in training who was blind to the assignment.

Means, ranges, and standard deviations were used for descriptive statistics. Categorical variables were compared using the *χ*^2^ test, continuous variables were compared using the independent samples t-test, and ranked data were compared using the rank sum test. Statistical analyses were performed with SPSS v. 26.0 (SPSS, Inc., Chicago, IL, United States), and significance levels were set as * *p* < 0.05; ** *p* < 0.01; *** *p *< 0.001.

## Results

A total of 58 patients (21 men and 37 women; mean age: 75.2 ± 9.2 years) were included, then divided into two groups: dual-mobility cup total hip arthroplasty (DMC group, *n* = 17) and internal fixation (IF group, *n* = 41). Surgeons recommended that all patients avoid excessive hip flexion, internal rotation, and adduction and perform rehabilitation exercises as early as possible. Patients who could not ambulate were advised to do simple exercises on beds.

The mean follow-up time for all patients was 12.3 months (range: 12–14 months). The two groups did not differ in sex, age, injury side, fracture kind, or muscle strength (Table 1). The average surgery length was 96.1 ± 18.3 min in the DMC group and 85.3 ± 24.9 min in the IF group (*p* = 0.139). The mean blood loss was 190.0 ± 171.5 ml and 19.8 ± 19.0 ml in the DMC and IF groups (*p* = 0.002). After surgery, only one sign of complication was detected in the DMC group, while 10 patients in the IF group experienced complications (*p* = 0.030). Additionally, the IF group had a higher incidence of fractures (40%) and vascular-related disorders (60%) (Table 2). Patients in the DMC group began rehabilitation exercises and walking earlier than those in the IF group.

**Table 1:** Baseline characteristics. Values are shown as Mean ± SD.

Variable	DMC group (*n* = 17)	IF group (*n* = 41)	*p*
Gender			0.926
Male	*n* = 6	*n* = 15	
Female	*n* = 11	*n* = 26	
Side			0.597
Left	*n* = 7	*n* = 20	
Right	*n* = 10	*n* = 21	
Fracture type			0.399
Garden I	*n* = 2	*n* = 7	
Garden II	*n* = 3	*n* = 15	
Garden III	*n* = 6	*n* = 10	
Garden IV	*n* = 6	*n* = 9	
Lovett muscle strength			0.790
0	*n* = 0	*n* = 1	
I	*n* = 1	*n* = 3	
II	*n* = 16	*n* = 37	
Mean age at surgery	73.2 ± 3.5 years	72.4 ± 11.2 years	0.782
Mean age at follow-up	75.5 ± 3.5 years	75.7 ± 12.1 years	0.948
Mean time of follow-up	34.7 ± 7.3 months	37.1 ± 8.5 months	0.458
Mean time to first exercise	3.2 ± 1.1 days	12.2 ± 5.6 days	< 0.001
Mean time to first ambulation	19.2 ± 4.8 days	78.7 ± 25.3 days	< 0.001

**Table 2:** Surgical data and complications. Values are shown as Mean ± SD.

	DMC group (*n* = 17)	IF group (*n* = 41)	*p*
Mean surgical duration	96.1 ± 18.3 min	85.3 ± 24.9 min	0.139
Mean Blood loss (ml)	190.0 ± 171.5 ml	19.8 ± 19.0 ml	0.002
Length of stay	5.4 ± 0.7 days	5.7 ± 0.9 days	0.349
**Complications**			
None	*n* = 16	*n* = 31	0.030
Cardiac failure	*n* = 0	*n* = 1	
Deep vein thrombosis	*n* = 0	*n* = 2	
Cerebral infarction;	*n* = 1	*n* = 2	
Cerebral.hemorrhage	*n* = 0	*n* = 1	
Fracture	*n* = 0	*n* = 4	

**Figure 1:**
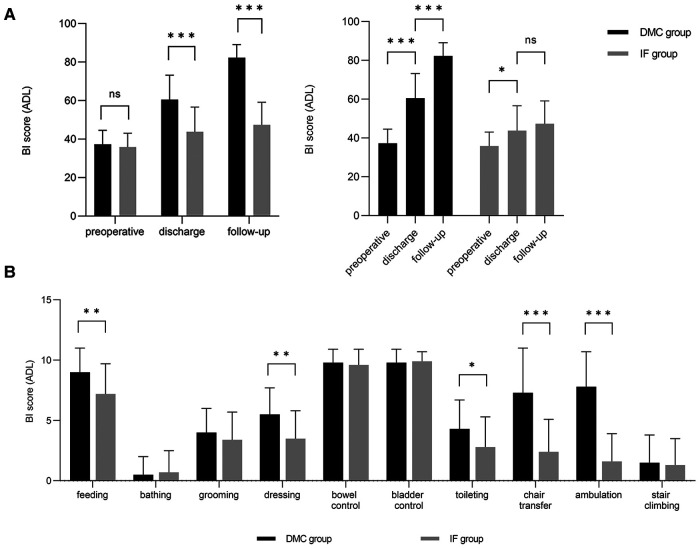
Scores for patient-related ADL assessed with the BI. The results of the DMC group cohort are shown in black and the IF group cohort are illustrated in grey. (**A**) Comparison and Change trend of ADL between DMC group and IF group at different time points. (**B**) Comparison between DMC group and IF group on subdimension scores of BI.

In the DMC group, patients had higher BI scores at admission, after surgery, and during follow-up than in the IF group (Figure 1). The BI scores significantly differed between preoperative, discharge and follow-up time in the DMC group (*p1, p2 *< 0.001). Meanwhile, preoperative and discharge scores significantly differed in the IF group (*p1* = 0.001) but not discharge and follow-up (*p2* = 0.187) (The *p1* value represents the *p*-value of the BI score between preoperation and discharge, while the *p2* value represents the *p*-value of the BI score between discharge and follow-up).

**Figure 2:**
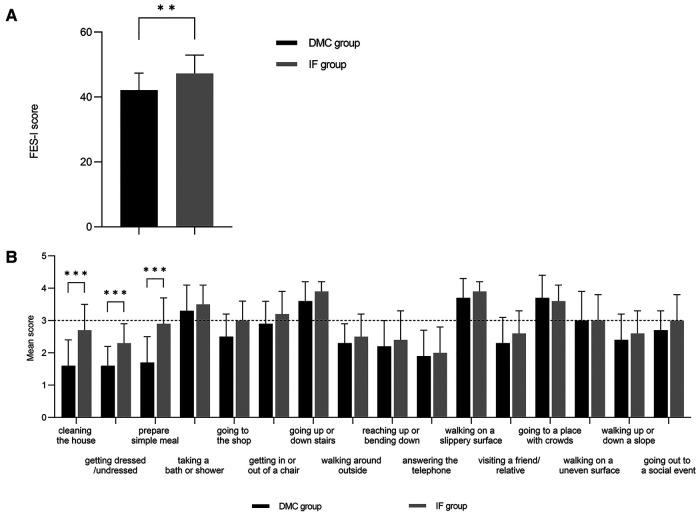
Scores for patient-related FOF assessed with the FES-I. The results of the DMC group cohort are shown in black and the IF group cohort are illustrated in grey. (**A**) Comparison of FES-I scores between DMC group and IF group. (**B**) Comparison between DMC group and IF group on subdimension scores of FES-I.

The mean FES-I score significantly differed between the two groups (DMC: 42.1 ± 5.3, IF: 47.3 ± 5.6; *p* = 0.002) (Figure 2A). In both groups, the mean score was > 3 for walking on a slippery surface (*p* = 0.013), going to a place with crowds, going up or down stairs, taking a bath or shower, and walking on an uneven surface. Besides, cleaning the house, getting dressed and undressed, and preparing simple meals were significantly different between groups (*p* < 0.001) (Figure 2B).

**Figure 3:**
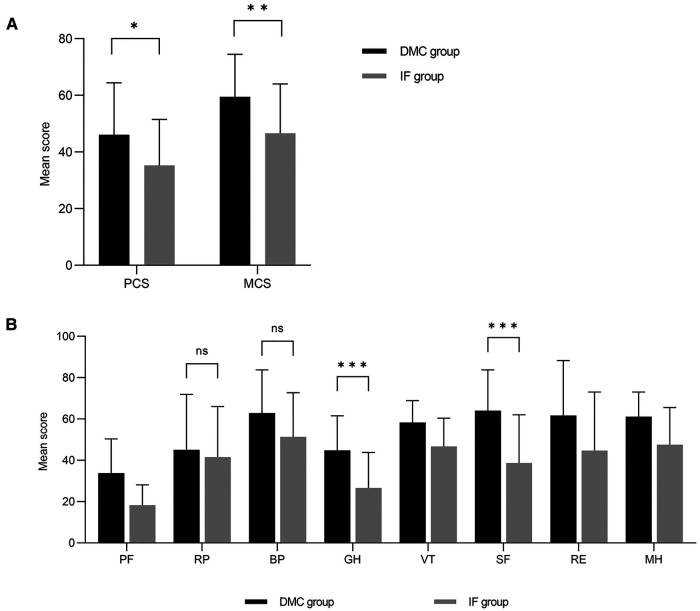
Scores for patient-related QOL assessed with the SF-36. The results of the DMC group cohort are shown in black and the IF group cohort are illustrated in grey. (**A**) Mean physical health component score (PCS) and mean mental health component score (MCS) assessed with the SF-36. (**B**) Comparison between DMC group and IF group on subdimension scores of SF-36.

The mean SF-36 physical health component score (PCS) was 46.1 ± 18.3 for DMC and 35.3 ± 16.1 for IF. The SF-36 mental health component score (MCS) was 59.5 ± 15.0 in the DMC group and 46.6 ± 17.6 in the IF group (Figure 3A). Patients in the IF group had poorer scores on both SF-36 components (*p *= 0.035 and *p* = 0.006, respectively), with mental scores lower than physical ones. Additionally, patients in the IF group had lower SF-36 subdomain scores (Figure 3B, Table 3).

**Table 3:** Subdimension scores of SF-36. Values are shown as Mean ± SD.

SF-36 Outcomes	DMC group (*n* = 17)	IF group (*n* = 41)	*p*
Physical health component (PCS)	46.1 ± 18.3	35.3 ± 16.2	0.035
Mental health component (MCS)	59.5 ± 15.0	46.6 ± 17.4	0.006
Physical function (PF)	33.8 ± 16.6	18.3 ± 9.8	0.001
Physical role (RP)	45.0 ± 26.9	41.5 ± 24.5	0.631
Bodily pain (BP)	62.9 ± 20.9	51.4 ± 21.3	0.058
General health (GH)	44.8 ± 16.7	26.6 ± 17.2	< 0.001
Vitality (VT)	58.3 ± 10.6	46.7 ± 13.7	0.002
Social functioning (SF)	64.0 ± 19.8	38.7 ± 23.3	< 0.001
Role emotional (RE)	61.7 ± 26.6	44.7 ± 28.3	0.031
Mental health (MH)	61.1 ± 11.9	47.5 ± 18.0	0.004

**Figure 4:**
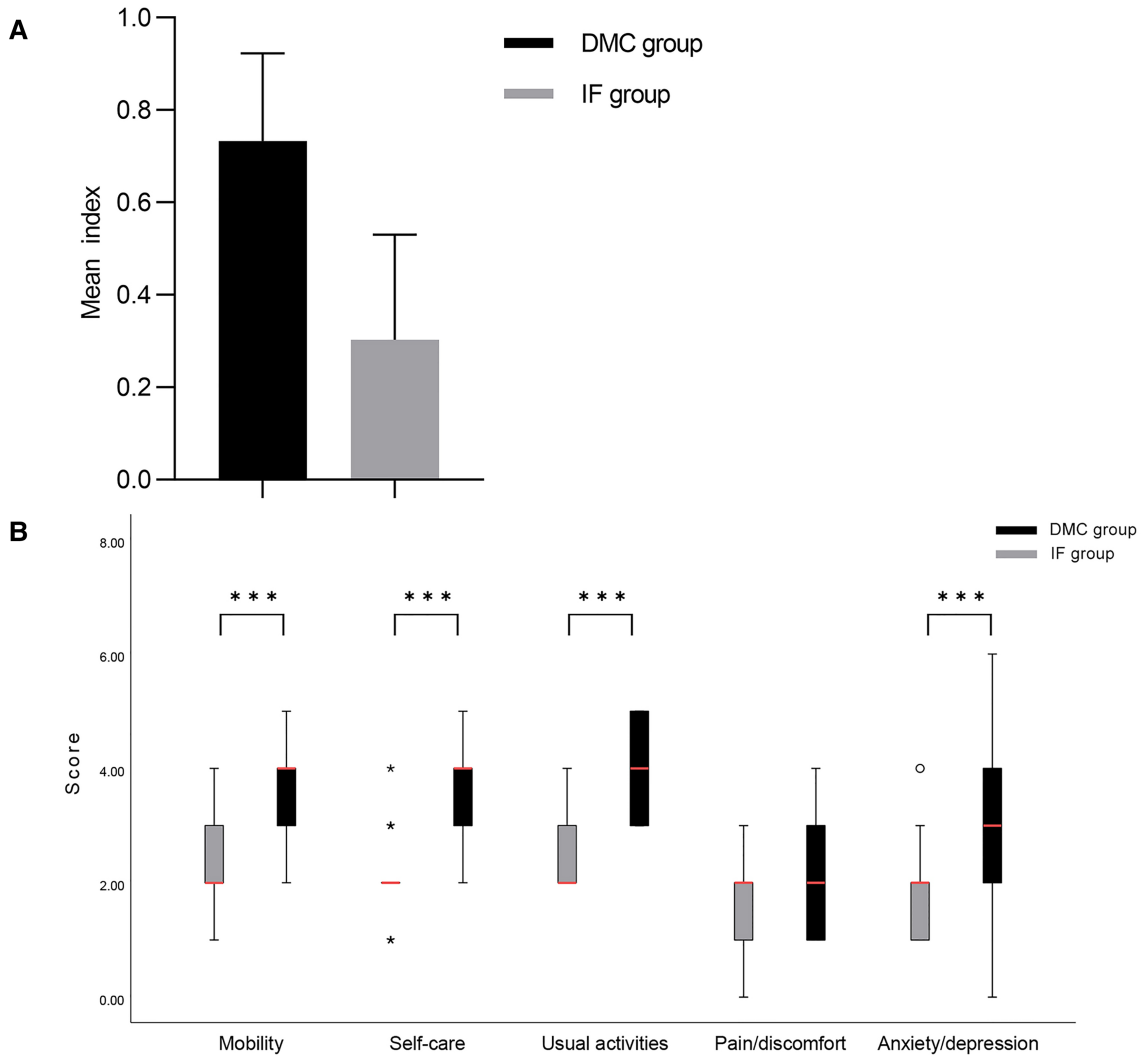
Scores for patient-related QOL assessed with the EQ-5D-5L. The results of the DMC group cohort are shown in black and the IF group cohort are illustrated in grey. (**A**) Comparison of EQ-5D-5L utility index between DMC group and IF group. (**B**) Comparison between DMC group and IF group on subdimension scores of EQ-5D-5L.

The mean EQ-5D-5L utility index was 0.737 ± 0.190 and 0.303 ± 0.227 for the DMC and IF groups (*p* = 0.035) (Figure 4A). At follow-up, The patients in the IF group had significantly worse walking, daily activities, and self-care capacity than those in the DMC group. A minimal difference was detected between the two groups for pain, and the DMC group had a much better psychological status than the IF group (Table 4, Figure 4B).

**Table 4:** Subdimension scores of EQ-5D-5L. Values are shown as Mean ± SD.

Dimensions of EQ-5D-5L	z/t	*p*
Mobility	5.139	< 0.001
Self-care	5.243	< 0.001
Usual activities	5.724	< 0.001
Pain/discomfort	1.326	0.096
Anxiety/depression	3.227	0.001

Furthermore, the QOL of patients in the DMC group was superior to the IF group at follow-up. The main differences were detected for the ability to perform daily activities, mental health, and function, while the pain did not differ.

## Discussion

HA and IF might not be the optimal therapeutic options for femoral neck fracture patients with grades < 3/5 lower limb muscular strength due to stroke. These patients are susceptible to dislocation after HA surgery due to a lack of muscle power in the lower extremities, and doctors usually choose IF (26–29). However, there are also shortcomings to IF, namely that patients must remain in bed for an extended period after surgery, which greatly affects their mobility and QOL. The DMC-THA may be a better solution. Some studies have shown that patients with neuromuscular diseases who undergo DMC-THA can quickly engage in rehabilitation training like individuals who have undergone HA, thereby achieving a lower risk of dislocation and complications, as well as a better quality of life (12, 30). However, for patients with severe lower limb weakness, postoperative rehabilitation is more difficult, and it is still unknown whether DMC-THA can maintain its advantages over IF. We found that DMC-THA improved the QOL by enhancing patients' physical and mental conditions.

Physical sickness and mobility are connected with QOL in older individuals (31, 32). THA has a higher surgical risk for patients with a history of cerebrovascular illness, resulting in a high risk of complications (33–35). However, we found no significant difference between the two groups regarding complications. Additionally, the proportion of postoperative problems was much lower in the DMC group (5.9%) than in the IF group (24.4%), which might be connected with early rehabilitation. In the short period after surgery, patients in the DMC group performed much better than those in the IF group regarding the BI score for dressing, toileting, flat ground transfer, and other low-intensity easy activities (Figure 1.3), indicating that low-intensity postoperative activity helps to lower complication risk (9, 36). We also demonstrated that the benefit of DMC-THA in enhancing mobility increased after discharge. At follow-up, BI scores were higher in the DMC group than in the IF group. Besides, the amplitude of the BI scores increased more in the DMC group relative to discharge than in the IF group, indicating that DMC-THA was more successful in recovering mobility and that its effects lasted longer. Despite receiving rehabilitation training, the patients in the IF group missed the “golden” early postoperative period. Consequently, continuous, early, uncomplicated home activities are essential for enhancing the postoperative QOL of femoral neck fracture patients with severe neuromuscular illness (10, 37).

The mental condition is a component of QOL evaluations (31). Previous research has shown that anxiety after surgery can decrease the QOL, consistent with our findings (38). The patient's anxiety remained for extended periods after surgery, mostly due to constraints in home-based activities. The FOF can confirm this idea, a psychological trauma after falls that can provoke further falls, anxiety, sadness, diminished physical activity, decreased QOL, and alterations in psychosocial function, impacting the rehabilitation of patients (39, 40). The FES-I results revealed significant differences in the mental status of indoor activities between the two groups: DMC was superior to IF, confirming our hypothesis that DMC-THA improves the QOL of patients with severe neuromuscular illness by enhancing home activities. In the FES-I outdoor activity section, no significant differences were detected, indicating that, even with DMC-THA, patients were still unable to cope with the complicated outdoor environment. Nevertheless, this did not influence the patients' general mental status. Therefore, by completing the reconstruction of indoor mental function, the QOL of older patients with impaired lower limb function can be considerably enhanced (41).

The SF-36 and EQ-5D-5L results demonstrated that patients' physical and mental health affect their QOL. In the SF-36 scale, the PCS and MCS indicate physical and mental status, respectively. Kimiko et al. (42) examined 8,333 patients and found that the MCS was an independent predictor of old age, whereas the PCS was not. We also found that mental status might be the primary reason for disparities in QOL between patients treated with DMC or IF. In the SF-36, significant differences were observed in the GH and SF subscales but not for RP and BP (Figure 3.2). Sim et al. (43) showed that GH, associated with the patient's self-perception level, was an independent factor influencing rehabilitation outcomes after hip fracture surgery. Patients with low GH scores feel gloomy about their situation, negatively affecting their attitude toward rehabilitation. Busija (44) found that changes in GH might not be noticeable in patients with minimal variation in health conditions before and after treatment, a phenomenon known as the GH ceiling effect. Consequently, GH differences indicated that DMC-THA could significantly improve patients' health status and confidence compared to IF, increasing patients' motivation to engage in rehabilitation exercises. SF is the social-relational dimension connected with physical and mental health. A previous study has shown that psychotherapy might greatly improve SF. Another study suggested the relevance of social ties in rehabilitating hip fracture patients (45–47). Our results supported these notions and revealed that optimism from early functional recovery might directly influence social connections in the elderly with significant mobility impairment. Therefore, psychological interventions during bed rest might improve the postoperative QOL of patients receiving IF. However, this topic should be further explored.

The RP and BP are unique subscales in the SF-36 and did not differ between the two groups. Changes in RP can persist over six months to two years with a single intervention under normal conditions (48). However, the effects of alternative interventions or more time on RP progression remain unknown. We believe that the lack of significant differences in RP was related to the limited sensitivity of the RP program to individual changes in patients, consistent with Busija et al. (39). Pain (BP) is one of the most significant elements in the QOL of patients, and postoperative pain might increase anxiety risk. For example, patients with femoral neck fractures tend to experience prolonged discomfort after IF, whereas those receiving arthroplasty do not, comprehending one of the most crucial reasons for choosing arthroplasty (38). However, the pain-related SF-36 and EQ-5D-5L subscales did not differ between the two groups, indicating that postoperative anxiety was not associated with pain in this study. These results might be partially related to old age and not operation type, which relevant studies have not previously demonstrated since healthy people have a decline in bone mass and are more susceptible to systemic discomfort as they age.

The EQ-5D-5L results demonstrated that the mean utility index of both groups did not meet the mean standard for the elderly in China (49), indicating that neither DMC-THA nor IF could fully restore the QOL, which was associated with the history of cerebrovascular disease. Nevertheless, the DMC group had a higher QOL than the IF group due to a greater recovery of daily activities and mental status, supported by additional scales. We found that the QOL might be associated with socioeconomic position, education, and the cost of in-home care during follow-up, influencing utility values (49).

However, our current study also has some limitations: (1) This was a retrospective analysis with a restricted number of eligible patients and various surgical procedures. In the future, high-quality research with larger samples and longer follow-up periods is required to corroborate our findings. (2) Although previous studies have established that the scales used here are equally powered, we cannot rule out variances in the results due to the telephone follow-up data collection for some patients. (3) Due to the history of cerebrovascular disease, we did not fully analyze the patient's postoperative problems in other aspects, which could have affected the QOL results. Thus, future studies should evaluate the impacts of other diseases on the QOL of these individuals.

## Conclusion

In summary, DMC-THA significantly improved postoperative QOL compared to IF in elderly stroke patients with severe neuromuscular dysfunction of the lower extremity. DMC-THA improved both physical and mental well-being. Enhancing patients' early, rudimentary motor performance improved physical and mental outcomes. Moreover, patients with complex illnesses tend to have low expectations for surgery. Early daily physiological function recovery increases their excitement for postoperative rehabilitation and enhances the treatment's efficacy.

## Data Availability

The raw data supporting the conclusions of this article will be made available by the authors, without undue reservation.
